# Potential Medicinal Fungi from Freshwater Environments as Resources of Bioactive Compounds

**DOI:** 10.3390/jof11010054

**Published:** 2025-01-10

**Authors:** Ilenia Cicero, Giulia Mirabile, Giuseppe Venturella

**Affiliations:** 1 Department of Agricultural, Food and Forest Sciences, University of Palermo, Viale delle Scienze, Bldg. 5, 90128 Palermo, Italy; ilenia.cicero01@unipa.it (I.C.); giuseppe.venturella@unipa.it (G.V.); 2 National Biodiversity Future Center (NBFC), Piazza Marina 61 (c/o Palazzo Steri), 90133 Palermo, Italy

**Keywords:** freshwater fungi, medicinal mushrooms, bioactive compounds, medicinal properties, macrofungi, microfungi

## Abstract

Owing to their nutritional, culinary, and nutraceutical, mushrooms are worldwide consumed and appreciated. Moreover, many of these mushrooms are also known as medicinal mushrooms since they possess several pharmacological properties attributable to a huge number of bioactive compounds derived from their sporophores. Several studies are available in the literature about in vitro and in vivo mechanisms of actions of such bioactive compounds. Most of these surveys are focused on macrofungi belonging to the genera *Pleurotus*, *Ganoderma*, or specific taxa such as *Agaricus bisporus*, *Agaricus blazei*, *Boletus eduliInonotus obliquus*, *Hericium erinaceus*, *Lentinula edodes*, and *Grifola frondose*. On the other hand, there is a lack of information on the under investigated ecological group of freshwater fungi. These fungi play a very important role in freshwater environments and some of them, belonging to Basidiomycota, are also edible and largely consumed. In this review we collected information about the medicinal properties of freshwater macro- and micromycetes. Among them, macrofungi, such as *Amanita vaginata*, *Armillaria mellea*, *Armillaria tabescens*, *Astraeus hygrometricus*, *Auricularia auricula-judae*, *Bjerkandera adusta*, *Bovista nigrescens*, *Calocybe gambosa*, *Candolleomyces candolleanus*, *Collybia dryophila*, *Coprinus comatus*, *Cyclocybe cylindracea*, *Hypsizygus ulmarius*, *Inonotus hispidus*, *Lactarius controversus*, *Lentinus tigrinus*, and *Schizophyllum commune*, observed in riparian habitat, and microfungi, such as *Penicillium aculeatum*, *P. chrysogenum*, and *Fusarium incarnatum*, isolated from aquatic plants, have been reported to have antimicrobial, anticancer, anti-inflammatory, antioxidant, antidiabetic, immunomoludatory, hypoglycaemic, and other pharmaceutical activities. Such fungal species are noteworthy since they represent an important quote of biodiversity to preserve their fundamental ecological role and a possible solution for different health problems for humans and animal farms.

## 1. Introduction

Mushroom consumption has increased in recent years thanks to their health-promoting effects rather than their nutritional properties. Indeed, many edible mushrooms have gained more attention as sources of bioactive compounds, including steroids, phenols, β-glucans, flavonoids, and alkaloids, that promote many pharmaceutical effects, such as antiviral, antibacterial, antifungal, anticancer, antidiabetic, hepatoprotective, cytotoxicity, anti-inflammatory, and hypocholesterolemic activities [1,2,3]. To date, it is estimated that among the 14,000 known fungal species, approximately 650 of them have shown in vitro or in vivo medicinal potential. Significant results have been obtained from terrestrial fungal species such as *Agaricus* spp., *Ganoderma lucidum* (Curtis) *P. Karst.*, *Boletus* spp., *Pleurotus* spp., *Lentinula edodes* (Berk.) Pegler, *Grifola frondosa* (Dicks.) Gray, and *Hericium erinaceus* (Bull.) Pers., all of which are well known for the presence of the above-mentioned bioactive molecules [3,4,5]. Compared with the large number (ca. 4000 taxa) of freshwater fungi [6,7], very little information about medicinal properties is available about a large percentage of these fungi and their related ecological groups. Freshwater fungi spend their entire life cycle, or part of it, in freshwater environments such as lakes, ponds, rivers, streams, wetlands, etc., or colonize submerged organs of plants in freshwater habitats. They belong to 13 fungal phyla, the most numerous of which are represented by Ascomycota, while a small number of species belong to Basidiomycota. Fungi require water for all life stages: spore germination, sporophore development, substrate degradation, nutrient uptake, reproduction, and spore dispersal. They degrade organic matter through the action of enzymes that need water to break down the substrate [6]. In aquatic environments, they act mainly as dead plants and animal biomass decomposers, but they are also involved in carbon and nitrogen cycles; they can act as plant or animal parasites or symbionts [7,8,9]. In a recent study by Mirabile et al. [9], according to the goals of the National Biodiversity of Future Center (NBFC) financed by the National Recovery and Resilience Plan (NRRP), an analysis of literature and field data reported several species of freshwater fungi isolated and observed in freshwater habitats of the Italian peninsula. This review aims to display information about the medicinal properties of freshwater fungi (both macro and micromycetes) as an important source of biodiversity useful for human and animal health.

## 2. Medicinal Properties of Freshwater Fungi

**Figure 1:**
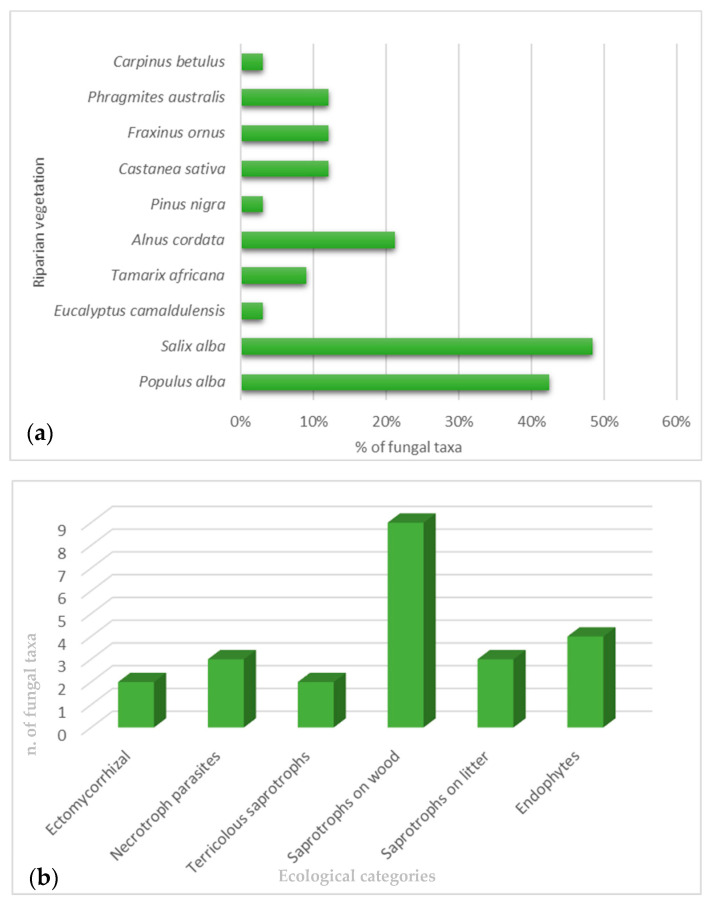
(**a**) Freshwater vegetation linked with the observed fungal taxa; (**b**) ecological category of freshwater macro- and microfungi.

A list of the medicinal properties of freshwater macromycetes observed in the riparian forests along the Italian peninsula, together with some freshwater micromycetes isolated from aquatic plants in the Italian territory [9,10], are reported here. In total, 19 macromycetes, growing both in terrestrial and freshwater habitats (*Amanita vaginata*, *Armillaria mellea*, *Armillaria tabescens*, *Astraeus hygrometricus*, *Auricularia auricula-judae*, *Bjerkandera adusta*, *Bovista nigrescens*, *Calocybe gambosa*, *Candolleomyces candolleanus*, *Collybia dryophila*, *Coprinus comatus*, *Cyclocybe cylindracea*, *Hypsizygus ulmarius*, *Inonotus hispidus*, *Lactarius controversus*, *Lentinus tigrinus*, *Schizophyllum commune*), and 3 micromycetes (*Penicillium aculeatum*, *P. chrysogenum*, and *Fusarium incarnatumi*) have been stated to have pharmaceutical activities (Table 1 and Table 2). The majority of macrofungi belonging to Basidiomycota phylum were found in the mixed riparian forest of *Populus alba* L. and *Salix alba* L., growing as saprotrophs on wood. In contrast, all the microfungi, belonging to Ascomycota phylum, were found in association with *Phragmites australis* (Cav.) Trin. ex Steud. as endophytes (Figure 1a,b).

### 2.1. Amanita vaginata *(Bull.) Lam.*

*Amanita vaginata* is an edible mushroom belonging to *Amanitaceae*. It is an ectomycorrhizal species that grows mostly in coniferous and hardwood forests. In riparian habitat, it was found associated with *Alnus cordata*, *Carpinus betulus*, and *Fraxinus ornus*. In the Asian region, particularly in India, this mushroom and its extracts have been deeply studied for their medicinal properties. The antibacterial activity of *A. vaginata* extracts was tested by Giri et al. [11]. The authors screened the antimicrobial potential of several wild edible mushrooms of West Bengal (India), among them *A. vaginata*. A methanolic extract was prepared and tested against pathogenic bacteria such as *Staphylococcus aureus*, *Proteus vulgaris*, *Bacillus cereus*, *Escherichia coli*, *Pseudomonas aeruginosa*, and *Bacillus subtilis* and against the pathogenic fungus *Candida albicans*. The antimicrobial activity was tested by using the disk diffusion method (500 µg/disk) and the results showed that the methanolic extract of *A. vaginata* exhibited inhibitory activity against three of the tested bacteria (*S. aureus*, *P. vulgaris*, and *E. coli*) and *C. albicans*. *A. vaginata* also have antioxidant properties due to their high content of polyphenols that can be useful for reducing the risk of heart diseases, but also anti-inflammatory, antiviral, antiallergic, anticarcinogenic and, immunomodulatory properties. Paloi and Acharya [12] analyzed the polyphenolic-rich fraction of *A. vaginata*, and found a significant level of total phenolic compounds (5.335 ± 0.28 µg gallic acid equivalent/mg of extract). Among the analyzed compounds, they found 0.81 ± 0.06 µg quercitin equivalent/mg of flavonoids, 1.0 ± 0.06, 0.6 ± 0.03, and 0.00035 ± 0.00005 µg/mg of extract of ß-carotene, lycopene, and ascorbic acid, respectively. As a result of the presence of these metabolites, *A. vaginata* showed a remarkable scavenging capacity against superoxide and hydroxyl radicals, chelating ability of ferrous ions, and a significant reducing power [12,13].

### 2.2. Armillaria mellea *(Vahl) P. Kumm.*

*Armillaria mellea* is an edible basidiomycete belonging to *Physalacriaceae* that acts as a necrotroph parasite in plants, causing a typical white rot that can lead the plant to death. In riparian habitat, it was observed to be associated with stumps of *Populus alba*, *Salix alba*, *Tamarix Africana*, and *Alnus cordata*. In some Asian countries, it is widespread for the treatment of many human diseases, such as insomnia, infantile convulsion, headache, microbial diseases, and neurasthenia, thanks to several bioactive compounds that have been isolated from its basidiomes. The presence of organic compounds such as β-glucans, sterols, peptides, sphingolipids, polyphenols, and other components make this fungus an important source of natural therapeutic substances [14,15]. Among the medicinal activities of *A. mellea*, their antioxidant capacity is among the most important. This capacity is due to the presence of phenols, carotenoids, flavonoids, and phenolic acid in *A. mellea* extracts. The tests that were carried out by Lung and Chang [16] and Erbiai et al. [17] using methanolic and hot water extract of *Armillaria* showed significant antioxidant capacity for concentrations of the extracts ranging from 0.5 to 20 mg/mL. Moreover, the extracts showed high reducing power, a DPPH radical-scavenging effect, a chelating ability on ferrous ions, and a scavenging effect on superoxide anion in a dose-dependent manner. The high efficiency in antioxidant properties was also confirmed by the EC50 values (effective concentration in which the antioxidant activity was 50%) of the extracts that were lower than 10 mg/mL.

Because of its antioxidant and antiapoptotic properties, the effect of polysaccharides obtained from *A. mellea* mycelium was also tested against Alzheimer’s disease. An et al. [18] tested the protective potential of polysaccharides extracted by using hot water at 80 °C from *A. mellea* mycelium on the induced HT22 cell apoptosis model and on Alzheimer’s disease induced in mice. *A. mellea* polysaccharides showed high levels of suppression of nuclear apoptosis, inhibited the accumulation of intracellular reactive oxygen, restored the potential of the mitochondrial membrane, and improved cell viability. Regarding Alzheimer’s disease induced in mice, *A. mellea* polysaccharides improved both horizontal movements during an autonomic activity test and endurance times in a rotarod test while decreasing the time of escape latency in a water maze test.

Methanolic extract of *A. mellea* also showed antibacterial and antifungal activity [19,20]. The extract was tested against the Gram-Positive bacteria *Staphylococcus aureus* (ATCC 6538), *Listeria monocytogenes* (NCTC 7973), a clinical isolate of *Bacillus cereus*, and *Micrococcus flavus* (ATCC 10240); Gram-Negative *Pseudomonas aeruginosa* (ATCC 27853), *Salmonella typhimurium* (ATCC 13311); and against the pathogenic fungi *Aspergillus niger* (ATCC 6275), *Aspergillus achraceus* (ATCC 12066), *Aspergillus versicolor* (ATCC 11730), *Aspergillus fumigatus*, *Trichoderma viride* (IAM 5061), *Penicillium funiculosum* (ATCC 36839), *Penicillium ochrochloron* (ATCC 9112), *Penicillium verrucosum* var. *cyclopium*, and four strains of *Candida albicans*. The extract showed antimicrobial activity, equally against pathogenic fungi and bacteria, with MIC values for inhibitory activity ranging from 3.12 to 15.00 mg/mL and bactericidal effect with MBC values ranging from 6.25 to 25.0 mg/mL.

Other compounds included in *A. mellea* basidiomes have been associated with anticancer effects. In particular, armillarikin and sesquiterpene aryl esters were found to be active against some cancer cells, causing leukemia and hepatoma [21,22,23].

### 2.3. Armillaria tabescens *(Scop.) Emel.*

*Armillaria tabescens* is a plant pathogen belonging to *Physalaciaceae* that can infect many woody plants such as fir, oak, eucalyptus, and almond trees [24]. In freshwater habitats, it was observed on stumps of *Populus alba* and *Salix alba* as necrotroph parasites.

Some bioactive compounds produced by *A. tabescens* have been investigated for their medicinal potential. Herath et al. [25] tested the antimicrobial activity of 7 *A. tabescens* chemical compounds obtained from its ethyl acetate extract: emestrin-G, emestrin-F, cephalosporolide-E, cephalosporolide-J, 6-O-(4-O-methyl-β-D-glucopyranosyl)-8-hydroxy-2,7-dimethyl-4H-enzopyran-one, decarestrictine-C2, brassicasterol and ergosterol. They were tested against fungal and bacterial human pathogens, including *Candida albicans* (ATCC 90028), *C. glabrata* (ATCC 90030), *Aspergillus fumigatus* (ATCC 204305), *Cryptococcus neoformans* (ATCC 90113), *Staphylococcus aureus* (ATCC 33591), *Pseudomonas aeruginosa* (ATCC 27853), *Escherichia coli* (ATCC 35218), and *Mycobacterium intracellulare* (ATCC 23068). The results showed that none of the 7 tested compounds had inhibitory effect against *A fumigatus*, *C. glabrata*, and *P. aeruginosa*. Only emestrin-F and emestrin-G showed antimicrobial activity. The first was active only against *M. intracellulare*, and the latter against *E. coli*, *S. aureus*, *C. albicans*, and *C. neoformans*.

Three extracts of *A. tabescens* (*n*-hexane, ethyl acetate, and methanol) were tested for their antioxidant activity [26]. Five methods were carried out to evaluate the antioxidant capability of the extracts: β-carotene bleaching method, DPPH radical scavenging activity, ABTS cation radical scavenging activity, metal chelating activity, and cupric reducing power. The results showed that the methanol extract exhibited a better antioxidant activity than the *n*-hexane and ethyl acetate extracts in the β-carotene bleaching method, DPPH radical scavenging activity, and ABTS cation radical scavenging activity. In detail, the methanol extract showed 90% inhibition of linoleic acid oxidation in the β-carotene assay at concentrations of 200 and 400 µg/mL, and 34.52 ± 0.39% and 88.48 ± 1.12% at concentrations of 400 µg/mL of antioxidant activity in the DPPH and ABTS assay, respectively. Regarding cupric reducing antioxidant capacity and metal chelating activity, the n-hexane extract resulted in the most active, with an absorbance (450 nm) value of 1.07 ± 0.01 and 57.13 ± 1.87% of inhibition at concentrations of 400 µg/mL, respectively.

### 2.4. Astraeus hygrometricus *(Pers.) Morgan*

*Astraeus hygrometricus* is a basidiomycete belonging to *Diplocystaceae*. It is commonly known as hygroscopic earthstar, barometer earthstar, or false earthstar for its aptitude for water-absorbing. It can open its rays, exposing its spore sac to increasing humidity, and close them in drier conditions. It is often recorded in association with *Quercus* spp. and *Pinus* spp. or growing as a symbiont with *Shorea robusta* G.f. [27,28]. In freshwater ecosystems, it is frequently associated with mixed riparian forests of *Tamarix africana*, *Salix alba*, and *Populus alba*. Many researchers have highlighted the presence of different bioactive compounds obtained from basidiomes of *A. hygrometricus*. This fungus is very appreciated for its medicinal properties, especially in India and China [29,30]. Among the phytochemicals with bioactive properties those produced by *A. hygrometricus* are known to be both high- and low-molecular-weight compounds. The first group belongs to polysaccharides and proteins, such as lectins, D-galactose, D-glucose, D-galacturonic, D-mannose, and L-fucose, while among the low-molecular-weight-compounds, we can find three triterpenes known as Astrahygol, 3-epi-Astrahygol, and Astrhygrone, steroids, and sesquiterpenoids, such as Astrakurkurol and Astrakurkurone [31,32,33,34,35].

Several authors reported a strong antioxidant activity of the extract of *A. hygrometricus*. In their survey, Bisaw et al. [36] recorded the positive effect of the ethanolic extract of *A. hygrometricus* for hydroxyl radical scavenging activity, β-carotene bleaching inhibition, superoxide, and DPPH scavenging activities, lipid peroxidation, and nitric oxide synthase activation properties, owing to the high presence of phenolic and flavonoid compounds in the extract. Also, the methanolic extract of this mushroom showed high antioxidant activities also linked to the presence of phenolic compounds such as protocatechuic acid, salicylic acid, ferulic acid, syringic acid, and anthralinic acid [30,37]. *A. hygrometricus* was also tested for its antimicrobial activity against several human pathogenic bacteria such as *Bacillus cereus*, *B. subtilis*, *Escherichia coli*, *Pseudomonas aeruginosa*, *Candida albicans*, *Staphylococcus aureus*, and *Proteus vulgaris* [37,38]. The methanolic extract was tested against the pathogenic bacteria by the disk diffusion method, showing antimicrobial activity against *P. vulgaris*, *E. coli*, *P. aeruginosa*, and *C. albicans* at a concentration of 500 µg/disk. The ethanolic extract of *A. hygrometricus* was also tested for its antimicrobial effect against *Leishmania donovani* promastigotes, a dangerous protozoan parasite causing visceral Leishmaniasis in humans. Lai et al. [35] were the first to isolate a new triterpene, from *A. hygrometricus* basidiocarp, named Astrakurkurone, that exhibited high promastigocidal activity against *L*. *donovani*. Subsequently, other researchers [39,40,41] discovered the mechanism of action of Astrakurkurone causing mitochondrial dysfunction in *L*. *donovani* and the capacity of this triterpene to induce the human cells to produce protective cytokines active against the parasite in both in vitro and in vivo tests in patients with visceral leishmniasis.

The ethanolic extract of *A. hygrometricus* also showed anti-diabetic activity at a dose of 500 mg/kg of body weight on alloxan-induced diabetic mice; hepatoprotective activity against CCl4 induced chronic hepatotoxicity; cardioprotective effect reducing the incidence of cardiac hypertrophy, myocardial infarction, and vascular death in cardiovascular compromised patients; anticancer activity against Ehrlinch’s ascites carcinoma cells in an animal model, apoptosis in tumor cells of mouse melanoma, HT-29 colon cancer, Dalton’s lymphoma, and sarcoma-180 [42,43,44].

Moreover, polysaccharides and proteins isolated from the aqueous extract of *A. hygrometricus* showed immunomodulator activity, stimulating T cells, B cells, and macrophages; amplifying phagocytic potential; cytokine production from splenocytes, thymocytes, and bone marrow cells of animal models [44,45,46].

### 2.5. Auricularia auricula-judae *(Bull.) Quél.*

*Auricularia auricula-judae*, also known as Jew’s ear, is a basidiomycete belonging to *Auriculariaceae* because of its ear-like shape and because it grows especially on elder wood (*Sambucus nigra*), the tree from which, according to the belief, Judas Iscariot hanged himself. It also grows on *Acer pseudoplatanus* L., *Fagus sylvatica* L., *Fraxinus excelsior* L., and *Euonymus europaeus* L. [47]. In freshwater ecosystems, it acts like a saprotroph on stumps and trunks of *Populus alba* and *Salix alba*.

Even if not widely consumed in East Asian countries, it has been frequently used as food and drugs for a long time thanks to the presence of several bioactive compounds with medicinal properties [48,49,50]. In their survey, Oli et al. [51] tested the antimicrobial potential of two protein extracts (tris buffer extract and warm aqueous extract) of *A. auricula-judae* against the pathogenic bacteria *S. aureus*, *B. subtilis*, *E. coli*, *P. aeruginosa*, *K. pneumoniae*, against the pathogenic yeast *C. albicans* and against the dermatophytes pathogens *Trichophyton schoenleinii*, *T. mentagrophytes*, *Microsporum gypseum*, and *M. ferugineum*. Results showed that both extracts exhibited antimicrobial effect against all the tested pathogens, except for *T. mentagrophytes*, *M. ferugineum*, and *M. gypseum*, with MIC values of 5 µg/mL for *S. aureus*, *B. subtilis*, *P. aeruginosa*, *K. pneumoniae*, and *C. albicans*, and of 2.5 µg/mL for *E. coli*.

Thanks to the production of polysaccharides, polyphenols, flavonoids, and melanin, *A. auricula-judae* shows a fundamental role in antioxidation processes against free radicals, preventing chronic diseases [52,53]. The antioxidant and anti-inflammatory activities of 30 varieties of *A. auricula-judae* were tested by Islam et al. [53]. Ferric reducing antioxidant power (FRAP, expressed as mmol of Fe^2+^ equivalents per 100 g of the sample), DPPH free radical scavenging (expressed as TE, Trolox equivalents, per g of sample), ABTS radical scavenging (expressed as TE, Trolox equivalents, per g of sample) and metal chelating ability (MCA, expressed as EDTA-2Na equivalents per g of sample) were carried out to test the antioxidant effect, and the anti-inflammatory activity was tested by the colorimetric protease inhibitory method. Results showed that FRAP values ranged from 0.53 mmol/100 g to 1.15 mmol/100 g; DPPH free radical scavenging activity values ranged from 1.50 µmol TE/g to 4.08 µmol TE/g; ABTS radical scavenging ability values ranged from 3.85 µmol TE/g to 10.04 µmol TE/g; and MCA ability values ranged from 1.59 µmol/g t3.50 µmol/g. These results indicate that the important antioxidant capability of *A. auricula-judae* strains can reduce Fe^3+^ to Fe^2+^ and inhibit the formation of ferrous and ferrozine complexes. Moreover, a test carried out to evaluate the anti-inflammatory activity by using the colorimetric protease inhibitory method revealed a high anti-inflammatory potential with IC50 values (anti-inflammatory activity of 50%, inhibiting concentration) ranging from 0.36 mg/mL to 0.50 mg/mL. In addition to possessing antimicrobial and antioxidant activities, *A. auricula-judae* seems to promote the skin wound-healing process thanks to the presence of bioactive polysaccharides. Mapoung et al. [54] tested this capability by in vitro and in vivo tests using a methanolic extract of *A. auricula-judae*. The in vivo tests were carried out on the HaCat cell line (human katinocystes) and primary human skin fibroblast isolated from an abdominal scar after a Cesarean practice. First of all, the effect of the *A. auricula-judae* extract was tested on cell proliferation, treating them with different concentrations of the fungal extract. Subsequently, the wound-healing activity was tested by horizontally scraping the monolayer confluent cells using a pipette tip and then washing them with PBS. Different concentrations of fungal extract ranging from 0.25 µg/mL were used. The effect of *A. auricula-judae* extract was also tested for cell migration and invasion and collage synthesis using the same extract concentrations. The results showed that both human fibroblast and keratinocyte increased proliferation, migration, and invasion process in a dose-dependent manner when treated with *A. auricula-judae* extract, reaching statistical significance at 20 and 25 µg/mL for both cells. Also, collagen synthesis was promoted by the treatment with fungal extract, demonstrating the high potential of in vitro, for *A. auricula-judae* to be used as a wounding-healing or a wound-dressing agents. With regard to in vivo tests, they were carried out on BALB/c male mice, six to eight weeks old, which were subjected to full skin excision by practicing circular wounds (5 mm in diameter). The mice were divided into three groups: one treated with 0.9% saline solution, and the others treated with 1% *w*/*v* or 2.5% *w*/*v* of fungal extract for 12 days. The results showed that the mice treated with the fungal extract showed a reduction in granulation tissue (for both of the used concentrations) and a thickening of the epidermis (for the group treated with 2.5% of fungal extract). Moreover, they showed an accumulation of fibroblast cells, a denser collagen network, and a more rapid wound closure rate if compared with the control group.

### 2.6. Bjerkandera adusta *(Willd.) P. Karst.*

*Bjerkandera adusta* is a basidiomycete belonging to *Meruliaceae*. It grows on dead wood, broad-leaved as a necrotroph. It can also act as a plant pathogen, causing white roots in living trees. Furthermore, it is widespread in America, Europe, and Asia. Thanks to the production of several enzymes that are able to degrade aromatic hydrocarbons, it is widely studied for its possible use in bioremediation and cosmetic applications (melanin decolorization) [55,56]. In freshwater environments, stumps and trunks of *Populus alba* and *Salix alba* were reported to be acting like a necrotroph parasite.

*B. adusta* basidiomes are rich in phenols, ergosterol, esters, squalene, and other bioactive compounds, suggesting that its cultivation could be useful for the production of secondary metabolites of medicinal interest [57]. In this regard, Soliman and El-Sayed [58] studied the potential of *B. adusta* as an antimicrobial agent. The ethanol extract of the fungus was tested against six pathogenic bacteria such as *E. coli* ATCC 25922, *P. aeruginosa* ATCC 7853, *S. aureus* ATCC 25923, *Proteus mirabilis*, *Micrococcus luteus*, and *S. pneumoniae*, and against the pathogenic yeast *C. albicans* ATCC 1031. The extract had an antimicrobial effect on all the pathogens except for *P. mirabilis*, with the diameter of the inhibition zone ranging from 10.0 ± 0.52 mm (*P. aeruginosa* ATCC 7853) to 22.0 ± 0.10 mm (*M. luteus*). De Oliveira et al. [59] evaluated the nutritional composition of *B. adusta* and its antioxidant activity. Four different extracts of *B. adusta* were prepared: hexane extract (EH), chloroform extract (EC), ethyl acetate extract (EAE), and methanolic extract (EMEOH). Their antioxidant potential was tested by measuring the DPPH radical scavenging activity, using the posphomolybdenum method, measuring the reducing power, and the thiobarbituric acid reactive substances (TBARS) analysis. The results showed that among the four tested extracts, only EAE (229.05 ± 1.3 µg·mL^−1^) and EMEOH (340.46 ± 2.0 µg·mL^−1^) presented DPPH radical scavenging activity; EAE (55.64 ± 2.92%) showed a better antioxidant activity than the control (ascorbic acid) in the phosphomolybdenum method; no statistically significant differences were found among the extracts in the reducing power analysis; and that EC and EH showed higher results than the control (BHT standard) in the TBARS analysis with lipid peroxidation values of 133.03% and 75.56%, respectively.

### 2.7. Bovista nigrescens *Pers.*

*Bovista nigrescens* is an edible basidiomycete belonging to *Lycoperdaceae*. It can be found in pasture land and grass in North and West Europe. In a riparian forest of *Salix alba*, *Populus alba*, and *Eucalyptus camaldulensis*, it grows as a saprotroph on litter.

The ethanolic extract of *B. nigrescens* was tested via disk diffusion assay for its antimicrobial properties against a wide group of pathogenic microorganisms: *Bacillus subtilis* ATCC 6633, *Enterococcus durans*, *E. faecalis* ATCC 29212, *E. faecium*, *L. monocytogenes* ATCC 7644, *S. aureus* ATCC 25923, *S. carnosus* MC1.B, *S. epidermidis* DSMZ 20044, *S. agalactiae* DSMZ 6784, *Enterobacter aerogenes* ATCC 13048, *E. coli* ATCC 25922, *E. coli* CFAI, *Klebsiella pneumoniae*, *Salmonella enteritidis* ATCC 13075, *S. infantis*, *S. kentucky*, *S. typhimurium* SL 1344, and *C. albicans* ATCC 10231. The extract was tested at three different concentrations: 400 µg/µL, 600 µg/µL, and 1000 µg/µL. Among the three tested concentrations, the extract at 400 µg/µL and 600 µg/µL had antimicrobial effects on *B. subtilis* ATCC 6633, *E. aerogenes* ATCC 13048, *E. coli* CFAI, *K. pneumoniae*, and *S. carnosus* MC1.B, while only the extract at 1000 µg/µL antimicrobial activity was also registered against *S. typhimurium* [60]. Bal et al. [61] tested both the antimicrobial and the antioxidant properties of *B. nigrescens* ethanolic and methanolic extract. To test the antimicrobial activity, an agar dilution test was carried out against *S. aureus* ATCC 29213, *S. aureus* MRSA ATCC 43300, *E. faecalis* ATCC 29212, *E. coli* ATCC 25922, *P. aeruginosa* ATCC 27853, *Acinetobacter baumannii* ATCC 19606, *C. albicans* ATCC 10231, *C. krusei* ATCC 34135, and *C. glabrata* ATCC 90030. Both extracts were tested at four concentrations (50, 100, 200, and 400 µg/mL). Ethanolic extract was active at 50 µg/mL against *S. aureus* ATCC 29213 and *S. aureus* MRSA ATCC 43300; at 100 µg/mL, it was active against *E. faecalis* ATCC 29212, *E. coli* ATCC 25922, *Acinetobacter baumannii*, ATCC 19606, *C. albicans* ATCC 10231, *C. krusei* ATCC 34135, and *C. glabrata* ATCC 90030; and against *P. aeruginosa* ATCC 27853, it was active at 200 µg/mL. Regarding methanolic extract, it showed antimicrobial activity against *S. aureus* ATCC 29213, *S. aureus* MRSA ATCC 43300, *A. baumannii* ATCC 19606, and *C. glabrata* ATCC 90030 at a 100 µg/mL concentration; against *E. faecalis* ATCC 29212, *E. coli* ATCC 25922, *C. albicans* ATCC 10231, and *C. krusei* ATCC 34135 at a 200 µg/mL concentration; and against *P. aeruginosa* ATCC 27853 at a 400 µg/mL concentration. The same extracts were also tested for their antioxidant activity by using the Rel Assay Diagnostic evaluating Total Antioxidant Status (TAS), Total Oxidant Status (TOS), and Oxidative Stress Index (OSI). The results showed that ethanolic extract showed the highest TAS value (4.140 ± 0.172 mmol/L), while methanolic extract showed the highest TOS (9.655 ± 0.160 µmol/L) and OSI values (0.261 ± 0.011 TOS/(TAS *×* 10)), demonstrating that *B. nigrescens* possess not only antimicrobial potential but also antioxidants properties.

### 2.8. Calocybe gambosa *(Fr.) Donk*

*Calocybe gambosa* is an edible basidiomycete, known as the St. George mushroom, belonging to *Lyophyllaceae*, and commonly growing in European grasslands. In freshwater ecosystems it was observed as a terricolous saprotroph in the riparian forests of *S. alba* and *P. alba*. Research carried out on *C. gambosa* basidiomes revealed a high content of flavonoids, phenolic compounds, ascorbic acid, tocopherols, β-carotene, lycopene, and the presence of other bioactive compounds that can be connected to some pharmaceutical effects [62]. In a recent study, the methanolic and aqueous extracts of *C. gambosa* were tested for their DPPH radical scavenging activity. The same extracts and a crude peptide extract were also tested for their cytotoxicity against human epithelial cervix adenocarcinoma (HeLa), epithelial lung carcinoma (A549), colon adenocarcinoma (LS174) cells, and human normal lung fibroblasts (MRC-5) [63]. Both extracts showed antiradical activity at the DPPH test, with IC50 1145.91 ± 88.60 µg mL^−1^ for aqueous extract and IC50 626.10 ± 25.20 µg mL^−1^ for methanolic extract. Regarding cytotoxicity, both methanolic and aqueous extracts did not show any activity against cancer and healthy cells (IC50 >200 mg mL^−1^). A cytotoxic effect was exhibited by the peptide extract in colon cancer cell line LS174 with an IC50 168.87 ± 9.72 µg mL^−1^. Other studies on the antioxidant activity of *C. gambosa* were carried out by other authors [64]. The methanolic extracts of the fungus was analyzed with several assays such as DPPH radical scavenging activity, ferricyanide/Prussian blue assay, TBARS assay and β–carotene/linoleate assay. Results showed that the antioxidant activity varied on the basis of the methodology. In particular, the best activity was showed with the ferricyanide/Prussian blue assay with an IC50 of 0.89 ± 0.02 mg/mL. The same extract was also tested for its antimicrobial activity against 8 pathogenic bacteria (*S. aureus*, *B. cereus*, *M. luteus*, *L. monocytogenes*, *P. aeruginosa*, *E. cloacae*, *S. typhimurium* and *E. coli*) using a modified microdilution method and against 8 pathogenic fungi (*Aspergillus ochraceus*, *A. fumigatus*, *A. niger*, *A. versicolor*, *Penicillium funiculosum*, *P. ochrochloron*, *P. verrucosum* var. *cyclopium*, and *Trichoderma viride*) by serial sub-cultivation of a 2 µL of the tested sample. The best antimicrobial effect was registered against *S. aureus* with MIC values of 9.30 mg/mL and MBC value of 18.60 mg/mL, among bacteria, and against *T. viride* and *A. versicolor*, among fungi, with MIC value of 10.94 mg/mL and MFC value of 43.75 mg/mL for both species.

### 2.9. Candolleomyces candolleanus *(Fr.) D. Wächt* and *A. Melzer*

The basidiomycete *Candellomyces candolleanus* belongs to *Psathyrellaceae*. It occurs in Europe, Asia, Africa, and North America. In the wild, it can act as facultative mycorrhizal fungus on *Poa annua* L. or as a saprotroph on cottonwood, elm, and beech trees. Its consumption is not widespread while in Iraq is traditionally used as medicinal food [65]. In freshwater habitats, it is reported as a saprotroph on dead wood stumps of *P. alba*, *S. alba*, *Tamarix africana*, and *A. cordata*. *C. candolleanus* sporophores produce a number of diterpenoids with significant antibacterial activity. Liu et al. [66] tested the antibacterial potential of two diterpenoids, Psathyrin A and B, extracted from *C. candolleanus*. The compounds were tested against *S. aureus*, *S. enterica*, and *P. aeruginosa* and MIC50 values were calculated. Results showed that both the compounds had antimicrobial effecst on *S. aureus* and *S. enterica* while there is no activity on *P. aeruginosa*.

Due to the capacity of ZnO nanoparticles to exhibit antibacterial properties against both Gram-Positive and Gram-Negative bacteria, Ali et al. [67] synthesized them from an aqueous extract of *C. candolleanus* for the first time. They tested the ZnO nanoparticles against *B. subtilis*, *B. meurellus*, *E. coli*, and *Acetobacter rhizospherensis* by disk diffusion assay in dark conditions and under UV-A light. The results showed that they affected all the tested bacteria in a dose-dependent manner in both conditions but the most sensitive were *E. coli* in dark conditions and *B*. *subtilis* under UV-A light.

Recently, Zhao et al. [68] discovered seven new guanacastane diterpenoids, produced by liquid fermentation of *C*. *candolleanus*, named psayamin (compound **1**), psathins A, B, C (compounds **4**, **5**, **6**), psathins D, E (compounds **7**, **8**), and psathin F (compound **9**) were tested for their acetylcholinesterase inhibitory activity, cytotoxic, and apoptosis activities. Among the seven tested compounds, psayamin showed the highest anti-acetylcholinesterase activity (IC50 at 37.3 μM), significant cytotoxicity against five tumor cells including those of lung cancer A549, leukemia HL-60, liver cancer SMMC-7721, colon cancer SW480, and breast cancer MCF-7 (IC50 ranging from 10.87 ± 0.24 to 15.96 ± 0.30 μM), and remarkably induced apoptosis against HL-60 in a dose-dependent manner.

### 2.10. Gymnopus dryophylus *(Bull.) Murril*

*Gymnopus dryophylus* is a basidiomycete that belongs to the *Tricholomataceae*. It grows mainly in leaf litter of deciduous and coniferous forests (forests of *P. alba*, *A. cordata*, *C. sativa*, *S. alba*, *Pinus nigra*) both in terrestrial and freshwater habitats. Very little information are known about its medicinal properties. In recent decades, Pacheco-Sánchez et al. [69] studied the inhibitory effects of a polysaccharide extracted from *Collybia dryophila* (the previous name of *G*. *dryophilus*) against the nitric oxide (NO) production induced by lipopolysaccharide (LPS) and gamma interferon (IFNγ) or induced only by LPS in EAW 264.7 cells (a murine macrophage cell line). They found out that the fungal polysaccharide significantly inhibited the NO production in a dose-dependent manner without a cytotoxic effect on vital cells and a direct effect on nitric oxide synthase gene expression.

### 2.11. Coprinus comatus *(O.F. Müll.) Pers.*

*Coprinus comatus* is a basidiomycete belonging to *Agaricaceae*. It is also known as the shaggy ink cap because its gills are initially white, then pink, and then finally black, producing a black liquid similar to ink, which is filled with spores. It grows in grasslands and meadows in Europe and North America, and in China, it is very appreciated as food and is widely consumed [70]. In the freshwater riparian forests, it grows as a saprotroph of the litter of *P. alba*, *S. alba*, *A. cordata*, *F. ornus*, and *Castanea sativa*. Different studies reported several physiological and beneficial effects of *C. comatus*. Organic extracts of some *C. comatus* were screened to test their antiandrogenic activity and their capacity to interfere with the androgen receptor, which is the major drug target of prostate cancer therapy [71]. Fungal extracts were prepared from 7 strains of *C. comatus* by using ethyl acetate (EA), hexane (H), chloroform (C), and ethanol (E) and tested against human MDA-kb2 breast carcinoma, DU-145, and PC-3 AR-independent and LNCaP androgen-dependent prostate cancer cell lines. The authors found that all *C. comatus* extracts inhibit AR by more than 60%. The strains CC542-C, CC734-H, and CC263-E showed the capacity to inhibit the proliferation of LNCaP cells with IC50 values below 50 µg/mL. These strains showed that lower effects were tested against the proliferation of PC-3 and MDA-Kb2 cell lines, demonstrating high selectivity toward the LNCaP cells. Moreover, CC734-EA, CC96-EA, and CC252-H extracts showed inhibitory activity against the proliferation of both LNCaP and PC-3 cell lines but were less effective against MDA-Kb2, confirming that *C. comatus* could be used as a natural antiandrogenic modulator for the treatment of prostatic cancer. Other anticancer activities of *C. comatus* were reported by Zhang et al. [72], which showed how a glycan-binding protein (Y3) isolated from the fungus had inhibitory effects against human T-cell leukemia in a dose-dependent manner, causing 90% of the Jurkat cells’ apoptosis.

Among the medicinal properties of *C. comatus*, its anti-diabetic activity was deeply investigated. Liu et al. [73] studied the effects of *C. comatus* polysaccharide extracts CCPF (extract obtained from fungal fragments) and CCPP (extract obtained from fungal powder). Hyperglycaemia was induced in male adult ICR mice that were then treated with different concentrations of each extract. Blood samples were obtained from treated and control mice to detect the glucose levels in the blood. The results showed that the mice group treated with a daily administration of CCPF at a concentration of 1000 mg/kg presented a significant reduction in the blood glucose level compared to the control group, while no significant effects were detected for CCPP extract. The anti-diabetic properties of *C. comatus* polysaccharides were also confirmed by Gao et al. [74] and Ratnaningtyas et al. [75]. In the first case, the authors investigated the effects of *C. comatus* mycelium polysaccharide (CMP) against the diabetic nephropathic in streptozotocin-induced mice. The treatment with CMP (400 mg/kg/d) significantly improved the insulin resistance of the mice by suppressing kidney dysfunction, inflammation, and renal oxidative stress. On the other hand, Ratnaningtyas and co-workers, tested on streptozotocin induced hyperglycemic rats by using an ethanolic extract of *C. comatus*. They measured the efficacy of the fungal extract by evaluating their glucose in the blood, insulin, glycosylated hemoglobin (HbA1c), dipeptidyl peptidase-4 (DPP-4), glucagon-like peptide-1 (GLP-1), and glutathione (GLS) levels. Experimental mice were treated with fungal extracts for 14 days using three different concentrations: 250, 500, or 750 mg/kg. At the dose of 750 mg/kg, rats’ blood glucose levels were reduced by 26.69% and DPP-4 by 6.97%, while the dose of 500 mg/kg reduced HbA1c by 4.30%, GLP-1 by 71.09%, and GHS by 11.19%, while it increased insulin levels by 13.83%.

*C. comatus* was also tested for its antioxidant properties against carbon tetrachloride-induced liver injury in rats [76]. *C. comatus* extract was orally administered at concentrations of 0.835, 1.67, and 3.34 g/kg/d for 42 days, after which they were euthanized for blood and liver collection and histopathological examination. The results showed that the treatments with fungal extracts improved the antioxidant capabilities in a dose-dependent manner, and had positive effects on tetrachloride-induced liver damage in rats, proved by a decrease in aminotransferase level in serum and lipid peroxidation intensity. Also, histological analysis confirmed the antioxidant and hepatoprotective effects of *C. comatus*.

### 2.12. Cyclocybe cylindracea *(DC.) Vizzini* and *Angelini*

*C. cylindracea* is a saprotroph basidiomycete belonging to *Strophariaceae*, also known as poplar mushroom. It is one of the most appreciated edible fungi and its worldwide cultivated. It grows on dead wood of broad-leaved trees, especially *Populus* spp., *Ulmus* spp., *Salix* spp., and *Sambucus* spp. In freshwater ecosystems, it was found on stumps of *P. alba* and *S. alba* riparian forests. In Asia, it is consumed for its antioxidant capability. In this regard, several studies have been carried out to highlight the antioxidant potential of this mushroom. Sevindik et al. [77] investigated the total antioxidant status (TAS), total oxidant status (TOS), and oxidative stress index (OSI) of *C. cylindracea* ethanolic extract. The TAS and TOS values reached 4.325 mmol/L and 21.109 µmol/L, showing that *C. cylindracea* has a rich antioxidant potential. Krüzsely et al. [78] investigated the scavenging activity of a β-carboline alkaloid isolated from the methanol extract of *C*. *cylindracea* by applying the DPPH radical scavenging assay. This compound exhibited a marked radical scavenging activity with EC50 values of 119.1 ± 1.2 µg/mL. Recently, Landingin et al. [79] tested and confirmed the scavenging activity of an ethanolic extract of *C. cylindracea* by DPPH radical scavenging assay.

### 2.13. Hypsizygus ulmarius *(Bull.) Redhead*

Also known as elm oyster or blue oyster mushroom, *H. ulmarius* is an edible mushroom belonging to *Lyophyllaceae*. It grows as a saprotroph or parasite, causing brown rot on elm, box elder, and beech in temperate forests of North America, Asia, and Europe. In the freshwater habitat, such species grow as a saprotroph on stumps of *P. alba* and *S. alba*. In Japan and China, it represents one of the most important consumed mushrooms thanks to its flavor, nutritional content, and medicinal properties [80,81]. Several studies reported the pharmaceutical potential of *H. ulmarius*. Greeshma et al. [82] evaluated the antioxidant, anti-inflammatory, and antitumor activities of the ethanolic extract of *H. ulmarius* sporophore and mycelium. The antioxidant activity was tested in vitro by DPPH radical-scavenging activity, hydroxyl radical-scavenging activity, inhibition of lipid peroxidation, nitric oxide (NO) scavenging activity, ABTS radical-scavenging activity, and ferric-reducing antioxidant power (FRAP). Both mycelium and sporophore extracts showed significant DPPH radical-scavenging activity at a concentration of 1000 µg/mL with a percentage of inhibition of 85% (sporophore) and 88.3% (mycelium). Similar percentages were also reached at the same concentration for the hydroxyl radical-scavenging activity, while the mycelium extract showed more potential at inhibiting lipid peroxidation in NO-scavenging activity and FRAP assay. On the contrary, the sporophore extract showed the highest percentage of inhibition at ABTS radical-scavenging activity with respect to mycelium extract. To test anti-inflammatory activity, Swiss albino mice were treated with formalin or carrageenan for the induction of chronic paw edema. Both extracts reduced the paw thickness at all the tested concentrations (1000, 500, and 250 mg/kg), with respect to the control, but mycelium extract showed higher activity than sporophore extract. The preventive and curative antitumor effect of the extracts against Dalton’s Lymphoma Ascites cell lines was also tested, and results showed that both extracts significantly reduced DLA-induced solid tumor and had a preventive antitumor effect in a dose-dependent manner, but mycelium extract showed, in general, a higher antitumor effect than sporophore extract.

Important pharmaceutical effects are attributed to *H. ulmarius* polysaccharides named HUP, HUP-1, and HUP-2, obtained by hot water extraction. The polysaccharides showed high antioxidant potential and high reducing power when they were tested for DPPH and ABTS radical scavenging activity [83,84,85,86]. HUP was also tested for liver protection against alcohol-induced liver damage in alcohol-intoxicated rats, showing that a pre-treatment with high or low doses of the extract significantly reduced both the levels of hepatic oxidative stress and the serum enzyme behaviors, restoring biochemical constituents levels and improving the serum lipid levels, the serum enzymatic levels, the and non-enzymatic antioxidants levels in liver [87]. The polysaccharides HUP-1 and HUP-2 were tested for in vitro anticoagulant and anticancer effects. Anticoagulant tests were carried out by measuring Prothrombin Time (PT), Activated Partial Thromboplastin Time (APTT), and Thrombin Time, showing that HUP-2 had a significant effect in the extension of APTT while HUP-1 considerably prolonged the three of them. Regarding anticancer activity, HUP-1 and HUP-2 were tested against the PC3 human prostate cancer cell line (HUP-2), and the proliferation of HeLA, HT29, HepG2, and PC3 (HUP-1) cell lines showing significant inhibitory effects and cytotoxic activity against all of them [84,86].

Other tests were carried out with the methanolic extract of *H. ulmarius* and its petroleum ether and ethyl acetate fractions, regarding antidiabetic and anti-inflammatory potential. The antidiabetic effects were evaluated through the in vitro inhibition of salivary α-amylase, salivary sucrase, and α-glucosidase activities, alongside the anti-inflammatory activity with the inhibition of enzyme lipoxygenase (LOX), myeloperoxidase (MPO), and cyclooxygenase (COX) enzymes. The results showed that even if the three tested *H*. *ulmarius* extracts had pharmaceutical effects, the ethyl acetate fraction had both the highest antidiabetic and anti-inflammatory activity compared to methanolic extract and petroleum ether fraction [81].

### 2.14. Inonotus hispidus *(Bull.) P. Karst.*

*Inonotus hispidus* is a brown-rot basidiomycete belonging to *Hymenochaetaceae*. It acts as a parasite or a facultative saprotroph on European and Chinese broadleaf trees such as *Morus alba* L., *Fraxinus mandschurica* Rupr., *Populus euphratica* Olivier, *Acer saccharum* Marshall, *Sorbus aucuparia* L., and *Ulmus minor* Mill. In freshwater habitats, it can be observed as a saprotroph on the trunks of *P. alba* and *S. alba*. In China, it has been used as traditional medicine for treating cancer, stomach problems, diabetes, and other diseases, and for this reason, it represents one of the mushrooms with the highest economic importance [87].

The anticancer activity of *I. hispidus* is widely investigated. Several monomers have been discovered to have anticancer effects. The compounds MBP (3,3′-methylene-bis [6-(3,4-hydroxystyryl)-4-hydroxy-2H-pyran-2-one) and HDE ((4S,5S)-4-Hydroxy-3,5-dimethoxycyclohex-2-enone), isolated from the methanolic extract of *I. hispidus* basidiomes, were tested for their inhibitory effect on the proliferation of HepG2, MCF-7, Hela, A549, and H22 cancer cell lines. The results show that the compounds inhibited the proliferation of all the cancer cell lines, but the highest inhibition activity was registered against HePG2 [88,89]. Another compound named WIH3, isolated from fermented mycelium of *I. hispidus*, was tested against Hep-3B, melanoma cells B16, Hela, and MCF-7 cancer cell lines, showing strong inhibitory effects at IC50 values of 37.39 µg/mL, 29.32 µg/mL, 47.03 µg/mL, and 58.01 µg/mL, respectively [90].

Another important pharmaceutical property of *I. hispidus* is its antioxidant and antimicrobial effect. Angelini et al. [91] carried out a comparative analysis of the antimicrobial and antioxidant activities of methanolic extracts obtained from sporophores and liquid-cultured mycelia of *I. hispidus*. The antioxidant activity was measured by the DPPH and β-carotene/linoleic acid assay. Compared to the control (Trolox), sporophore and mycelia extracts showed an activity of 17.2 and 22.1%, respectively, in the DPPH assay, while in the β-carotene/linoleic acid assay, it showed activity values of 15.44 and 11.44%, respectively, compared to the control activity (BHT, butylated hydroxytoluene). The antimicrobial effect was tested against *P. aeruginosa* (ATCC 15442), *E. coli* (ATCC 10536), *Salmonella typhi*, *S. aureus* (ATCC 6538), *B. cereus* (ATCC 12826), *C. albicans* (YEPGA 6183), *C. tropicalis* (YEPGA 6184), *A. tubingensis* (PeruMicA 21), and *A. minutus* (PeruMica 22). The activity of basidiomes extract was higher than mycelia extract, with MIC values ranging from 0.17 to 1.71 μg mL^−1^.

### 2.15. Lactarius controversus *Pers.*

*Lactarius controversus* is a basidiomycete belonging to *Russulaceae* which is widespread in Europe and grows in symbiosis with *Salix* spp., and in North America; it is associated with *Populus* spp. and *Salix* spp. In riparian environments, it grows as ectomychorrhizal in the mixed forests of *S. alba*, *P. alba*, *A. cordata*, *F. ornus*, and *C. sativa*. In Europe, due to its acrid taste, it is not considered edible, but in Serbia and Turkey, it is widely consumed for its beneficial effects [92,93].

The ethanol and water extracts of *L. controversus* revealed radical scavenging effects on DPPH, OH, NO, and SOA radicals and FRAP potential. In particular, the water extract exhibited the highest DPPH radical scavenging effect (IC50 219.37 ± 5.7 µg ml–1), while the ethanol extract showed the highest FRAP capability (10.93 ± 0.9 mg ascorbic acid equivalents/g extract dry weight). The same extracts were tested for their antiproliferative activities against the human breast cell line MCF 7, showing that the ethanolic extract had better antitumor activity than the water extract [92]. The antioxidant effect of *L. controversus* was also registered for its methanol extract. The activity was evaluated by reducing Mo(VI) to Mo(V), linoleic acid peroxidation inhibition, power, metal chelating, superoxide anion scavenging, free radical scavenging, hydrogen peroxide scavenging, and peroxide scavenging activity, showing in all the assays their highest values at concentrations of 100 µg/mL [93]. Moreover, the hexane extract and methanolic extract of *L. controversus* were shown to possess antimicrobial activity. Hexane extract was tested against *S. aureus* ATCC 25922, *B. subtilis* ATCC6633, and *E. coli* ATCC25923 and showed inhibitory activity only against *S. aureus* ATCC 25922 at MIC and MBC concentrations of 3.12 mg/mL and 6.25 mg/mL, respectively. The methanolic extract was tested against *S. aureus* ATCC 6535, *B. cereus* ATCC 7064, *S. epidermidis* ATCC 12228, *E. coli* W3110, *P. aeruginosa* ATCC 27853, *E. aerogenes* ATCC 13048, and *C. albicans* ATCC 10231 as well as clinical isolates of *E. coli*, *P. aeruginosa*, *P. vulgaris*, *E. aerogenes*, *Acinetobacter baumanii*, *Morganella morganii*, Methicillin-resistant *S. aureus*-MRSA, Methicillin.resistant coagulase (-) *Staphylococcus*–MRKNS, showing an inhibition zone > 15 mm for *E. coli* W3110, *P. aeruginosa* ATCC 27853, *B. cereus* ATCC 7064, *S. aureus* ATCC 6535, and against the clinical isolates *P. aeruginosa*, *P. vulgaris*, and *M. morganii* [92,93].

### 2.16. Lentinus tigrinus *(Bull.) Fr.*

*Lentinus tigrinus* belonging to *Polyporaceae*, is a wood-rotting basidiomycete growing on fallen logs. It acts like a saprotroph on stumps of *P. alba* growing in riparian forests. Its strong aroma and taste make it widely used as a culinary mushroom [94]. Several surveys pointed out the pharmaceutical potential of this mushroom and its use as a functional food. The hot-water extract of *L. tigrinus* showed hypoglycemic activity in diabetic mice, lowering the glucose levels in their blood by 26.9% (value significantly comparable to the control) at concentrations of 100 and 250 mg/kg at the third week of treatment [95]. Hot water, ethanol, and acetonitrile extracts showed antibacterial effects against *S*. *aureus* by using a paper disk diffusion assay, with a diameter of inhibition zone of 8, 12.7, and 9.48 mm, respectively [95,96]. The antimicrobial effect of other extracts of *L*. *tigrinus* was confirmed by Sevinkid et al. [97]. In their study, they tested ethanol, methanol, and dichlorometane extracts against *S. aureus* ATCC 29213, *S. aureus* MRSA ATCC 43300, *E. faecalis* ATCC 29212, *E. coli* ATCC 25922, *P. aeruginosa* ATCC 27853, *Acinetobacter baumannii* ATCC 19606, *C. albicans* ATCC 10231, *C. krusei* ATCC 34135, and ATCC 13803, and *C. glabrata* ATCC 90030, by using the agar dilution method. The ethanolic extract exhibited the highest level of inhibition against all the tested pathogens except for *A. baumannii* against which none of the extracts showed inhibitory activity. On the other hand, methanol and dichloromethane extracts showed antimicrobial effects against *S. aureus*, *E. faecalis*, *C. albicans*, *C. glabrata*, and *C. krusei*, but not against *E. coli*, and *P. aeruginosa*.

*L. tigrinus* was also investigated for its antioxidant properties. Acetonitrile and hexane extracts showed the DPPH radical scavenging activity of 39.2% (EC50 value 637.75 mg/mL) and 35.5% (EC50 value 710.23 mg/mL), while tested for antioxidant status (TAS), total oxidant status (TOS) and oxidative stress index (OSI) carried out direct on sporophores demonstrated that *L. tigrinus* TAS, TOS, and OSI values were 1.748 ± 0.071, 19.294 ± 0.237, and 1.106 ± 0.031, respectively [96,97].

For the first time, the anticancer potential of a soluble protein fraction (LTPp) of *L. tigrinus* was also reported [98]. LTPp exhibited an antiproliferation effect against both MCF-7 and PC3 cancer cell lines, also inducing modification in their morphology. Moreover, LTPp at a concentration of 70 μg/mL was shown to kill approximately 45 and 70% of MCF-7 and PC3 cells, respectively, inducing cell apoptosis.

### 2.17. Pleurotus cornucopiae *(Paulet) Quél.*

*Pleurotus cornucopiae* is a saprophytic basidiomycete belonging to *Pleurotace*. It is often found on stumps and fallen trunks of oak, elm, and other broad-leaved species. It is widespread in Europe and China, where it is one of the most consumed mushrooms belonging to the genus *Pleurotus*. In freshwater ecosystems, it is reported as a saprotroph on dead wood of *P. alba* stumps. Several studies have reported on the medicinal properties of this species [3,5]. In a study conducted by Wang et al. [99], it was isolated from *P. cornucopiae* and two novel sesquiterpenes named pleurospiroketals A and E showed antioxidant activity, inducing the inhibition of nitric oxide productional lipopolysaccharide-activated macrophages (IC50 values ranging from 6.8 to 20.8 µM). The antioxidant activity of *P. cornucopiae* was also demonstrated by Zhang et al. [100], who isolated three intracellular zinc polysaccharides from the mushroom. These polysaccharides had action in the upregulation of superoxide dismutase, GSH peroxidase, and catalase, also reducing lipid peroxidation within in vivo tests. Antioxidant properties of *P. cornucopiae* were also investigated by Landingin et al. [79]. The radical scavenging activity of its ethanolic extract (concentration 1000 ppm) was measured via DPPH assay, recording a value of 41.75%. Tanaka et al. [101] carried out a double-blind, placebo-controlled human clinical trial and demonstrated the ability of *P. cornucopiae* to regulate the immune system. They evaluated the levels of serum cytokine involved in the regulation of the immune systems and demonstrated that *P. cornucopiae*, administered for 8 weeks, increased the levels of interferon (IFN-γ) and interleukin IL-12 in serum.

Some medical activities of *P. cornucopiae* are also related to its laccase production. A novel laccase isolated from broth cultivation of *P. cornucopiae* exhibited an inhibitory effect against the cancer cell lines HepG2 (hepatoma cells) and MCF-7 (breast cancer cells), with IC50 values of 3.9 and 7.6 µM. Moreover, it showed the inhibition of the activity of HIV-I reverse transcriptase with an IC50 value of 3.7 µM [102].

### 2.18. Pleurotus ostreatus *(Jacq.) P. Kumm.*

*Pleurotus ostreatus* is one of the most common edible mushrooms. It belongs to *Pleurotaceae*, and it usually can be found as a saprotroph and primary decomposer on deciduous trees, acting as a white rot wood decay fungus; it can also be found in freshwater ecosystems as saprotroph on *P. alba* stumps. It can grow in many places, and it can also be easily cultivated on straw or other media. Furthermore, it is consumed all over the world, both for its nutritional and medicinal value. This mushroom, indeed, possesses several pharmaceutical properties [3]. *P. ostreatus* extract showed a stimulatory activity against the catalase gene expression and a reduction in the incidence of free-radical-induced protein oxidation in rats, reducing age-related disorders [103]. Ethanolic extract and carbohydrate components, mainly β-glucans, showed antioxidant activity in vitro and in vivo in superoxide radicals, scavenging hydroxyl, lipid peroxidation, and reducing power on ferrous ions [104,105]. Cao et al. [106] demonstrated that polysaccharides extracted from *P. ostreatus* mycelium had both in vitro and in vivo antitumor properties against BGC-823 cells (human gastric cell lines), inhibiting the proliferation by 35.6% at a concentration of 400 mg/mL. Using *P. ostreatus* extracellular filtrate, El Domany et al. [107] synthesized gold nanoparticles to test them against the human cancer cell line HepG2, the prostate cancer cell line PC3, and the human colon cancer cell line HCT-116, demonstrating that *P. ostreatus* nanoparticles significantly reduced the viability of HepG2 and HCT-116 by 33.5% and 22.7%, respectively. Ethanol extracts of *P. ostreatus* named CT-WS, CP-WS, CT-LcS, and CP-LcS were tested for their antimicrobial properties against *E. coli* ATCC 10536, *E. coli* PeruMycA 2, *E. coli* PeruMycA3, *B. cereus* PeruMycA 4, *P. aeruginosa* PeruMyc 5, *B. subtilis* PeruMyc 6, *Salmonella typhy* PeruMyc 7, *S. aureus* ATCC 6538, *C. albicans* YEPGA 6183, *C. tropicalis* YEPGA 6184, *C. albicans* YEPGA 6379, *C. parapsilopsis* YEPGA 6551, *Arthroderma crocatum* CCF 5300, *A. curreyi* CCF 5207, *A. gypseum* CCF 6261, *A. insingulare* CCF 5417, *A. quadrifidum* CCF 5792, *Trichophyton mentagrophytes* CCF 4823, *T. mentagrophytes* CCF 5930, *T. rubrum* CCF 4933, *T. rubrum* CCF 4879, and *T. tonsurans* CCF 4834. All extracts showed antimicrobial effects at MIC values ranging from 6.25 to 200 µg/mL, and the highest levels of inhibition were registered for CT-LcS and CP-LcS against yeast; regarding bacteria, the highest activity was registered for CP-LcS [104]. Methanol extracts of *P. ostreatus* cultivated on different substrates were tested against *E. coli*, *S. aureus*, *C. albicans*, and *Cryptococcus neoformans*, showing inhibitory activity against all of them with MIC values ranging from 0.08 mg/mL to 2.5 mg/mL. However, *P. ostreatus* growing on sugarcane bagasse showed the highest antimicrobial activity against *E. coli*, also demonstrating that the growth substrate influences fungal medicinal properties [108]. Methanol and ethyl acetate extracts obtained from *P. ostreatus* sporophores significantly decreased blood glucose levels of streptozotocin-treated diabetic rats after two weeks of treatment by oral administration (200 mg/kg). Moreover, the administration of both methanol and ethyl acetate extract significantly reduced blood levels of total cholesterol by 16.92 and 26%, triglycerides by 17.33 and 29.33%, low-density lipoprotein (LDL) by 17.3 and 20%, while increasing the levels of HDL-cholesterol by 17.94 and 30.76%, respectively [109]. Since one of the strategies for the controlling of diabetes mellitus is to inhibit carbohydrate-digesting enzymes, such as α-amylase and α-glucosidase, the water/ethanol of *P. ostreatus* was tested for this purpose. Five different concentrations were tested, 50, 100, 150, 200, and 250 μg/mL and the results showed that the percentage of inhibition ranged from 14.65 ± 1.94 to 62.55 ± 1.07% for α-amylase and from 18.35 ± 1.70 to 82.54 ± 0.88% for α-glucosidase, with dose-dependent inhibitory effect [110].

### 2.19. Schizophyllum commune *Fr.*

*Schizophyllum commune* is a saprotroph species belonging to *Schizophyllaceae*. It usually grows on decaying trees, especially after rainy seasons. In freshwater habitats, it was recorded on the stumps and dead trunks of *S. alba* and *P. alba*. Its wide distribution and its pharmaceutical potential make it a globally consumed food supplement, but also an important source for industrial applications and cosmetics [111]. Among the medicinal properties of *S. commune*, its antioxidant potential, due to the high presence of phenolic compounds, is one of the most investigated [112]. Studies about the influence of raw materials used for *S. commune* cultivation on its antioxidant activity were examined by Basso et al. [113]. Pine sawdust (PS), grape residue (GR), cotton cake (CC), and jatropha seed cake (JK) were used as substrates of cultivation, and antioxidant activity was evaluated via DPPH radical scavenging assay. The results showed that *S. commune* samples grown on CC showed the best antioxidant activity (58.15 ± 0.86 DPPH % scavenging), probably due to the highest phenolic content (291.51 ± 1.83 mg GAE/100 g mushroom). Mišković et al. [114] tested submerged cultivated mycelia and fermentation broth of ethanolc and polysaccharide extract from two different *S. commune* strains (Italian, IT and Serbian, SRB) to determine their acetylcholinesterase (AChE) inhibitors production (important agents against Alzheimer’s), as well as their antioxidant and antibacterial activity. Polysaccharide extracts of SRB strains from submerged cultivated mycelia showed the highest AChE activity with IC50 values of 79.73 ± 26.34 µg/mL after 28 days of fermentation and significant antioxidant activity. Only two extracts showed antibacterial activity, and both came from the SRB strain. In particular, the highest antimicrobial activity was registered for the sporophores extract MIC and MBC value < 0.31%) against *E. coli*, *B. cereus*, and *S. aureus*. Hexane, chloroform, ethyl acetate methanol, and hot water extracts of *S*. *commune* were evaluated for their scavenging activity. On the other hand, crude polysaccharide was tested for hepatoprotective activity on human hepatoma-cells (HepG2) after hydrogen peroxide-induced toxicity. All the extracts showed DPPH radical scavenging activity with IC50 values of 0.57 ± 0.02 mg/mL, 0.40 ± 0.01 mg/mL, 0.36 ± 0.00 mg/mL, 0.32 ± 0.00 mg/mL, and 1.00 ± 0.05 mg/mL, respectively, showing a positive correlation between DPPH scavenging activity and the content of phenols and flavonoids. Regarding hepatoprotective activity, polysaccharide extract inhibited lipid peroxidation while increasing glutathione levels against hydrogen peroxide-induced damage [115]. Al-Azad and Ping [116] tested the antioxidant and antibacterial activity of aqueous, methanol, ethanol, and acetone extracts of *S. commune*. The antioxidant activity was tested by DPPH assay in which ethanol extract showed the highest scavenging activity (97.19%). The antimicrobial activity was verified in the three marine bacteria *Vibrio harveyi*, *V. parahaemolyticus*, and *V. anguillarum* by MIC determination. All the extracts showed the highest inhibitory activity against *V. harveyi* with MIC value lower than 1.25 mg/mL, while inhibitory activity were registered at MIC values of 2.5 mg/mL and 5 mg/mL for *V*. *parahaemolyticus* and at 5 mg/mL for *V*. *anguilarium*.

### 2.20. Penicillium aculeatum *Raper and Fennell*

*Penicillium aculeatum* is a mold belonging to *Trichocomaceae*. Very little information has been reported in the literature about it and its ecological role: in freshwater environments, it was isolated as an endophyte from the leaves and roots of *Phragmites australis*. This mold is significant in the production of pigments and other molecules with pharmaceutical properties. The broth and the mycelial ethyl acetate extract of *P. aculeatum* showed antimicrobial activity against *S. aureus* ATCC 25923 (MIC value of 200 and 128 µg/mL, respectively). Only the second one showed antimicrobial activity also against *Criptococcus neoformans* ATCC 90113 (MIC value 200 µg/mL). Moreover, it also has cytotoxic activity against KB cell lines (oral cavity cancer cell lines) with an IC50 value of 27.56 µg/mL. Antimicrobial activity was registered for mycelial hexane extract against *C. neoformans* and *Mycobacterium tuberculosis* H37Ra (MIC values of 200 and 50 µg/mL, respectively), together with the antimalarial effect, and the cytotoxic effect against KB and MCF-7 (IC50 values of 3.75, 21.14, and 35.74 µg/mL, respectively). In addition, nine secondary metabolites were isolated from the broth extract, and some of them exhibited antimicrobial and cytotoxic activity. In particular, penipurdin A showed an inhibitory effect against *M. tuberculosis* with an MIC value of 25 µg/mL. Altenusin showed a moderate inhibitory effect against *S. aureus* with an MIC value of 32 µg/mL and a moderate cytotoxic effect against African green monkey kidney fibroblast [117]. The ankaflavin, a pigment extracted from *P. aculeatum*, was tested for cytotoxic activity against MCF-7, HCT116, and PC-3 cell lines. While it showed very limited toxicity against MCF-7, significant results were obtained against HCT116 and PC-3 cell lines with IC50 values of 162 µg/mL and 85 µg/mL, respectively [118]. Hawas et al. [119] isolated two new sulfonyl metabolites from the ethyl acetate extract of *P. aculeatum* called pensulfonoxy and pensulfonamide. Ethyl acetate extract showed antibacterial activity against *E. coli* (diameter of inhibition zone of 20.5 mm), while pensulfonamide showed not only an inhibitory effect against *C. albicans* (diameter of inhibition zone of 18 mm) but also potent cytotoxicity against MCF-7 cell line (IC50 2.18 µM). In contrast, pensulfoxony only showed a mild cytotoxic effect against the HTC-116 cell line (IC50 5.23 µM).

### 2.21. Penicillium chrysogenum *Thom*

*P. chrysogenum* is a micormycetes belonging to *Thricomaceae*. It is commonly found in temperate and subtropical regions both in soil and in dead plant material, and it is also known as a post-harvest contaminant of fruit, vegetables, and grains. In freshwater ecosystems, it was associated as endophyte with roots of *P. australis*. *P. chrysogenum* represents one of the most studied species of the genus *Penicillium* for its capability to produce penicillin and other antibiotic compounds [120]. Holzknecht et al. [121] reported the anti-*Candida* properties of the antifungal protein C (PAFC) extracted from *P. chrysogenum* that was able to kill the planktonic cell and reduce the metabolic activity of sessile cells in the biofilms of two different strains of *C. albicans*. Orfali et al. [122] tested the antimicrobial activity of several dihydroisocumarins isolated from *P*. *chrysogenum* against the pathogenic bacteria *S. aureus*, *B. licheniformis*, *Escherichia fergusonii*, *Enterobacter xiangfagensis*, and *P. aeruginosa*. These compounds showed high antibacterial activity against *S. aureus* and *B. licheniformis* with MIC values ranging from 0.8 to 21.6 μg/mL. Recent studies reported the cytotoxic potential of *P. chrysogenum*. Niu et al. [123] tested different compounds isolated from *P. chrysogenum* against BEL-7402, BIU-87, ECA109, Hela-S3, and PANC-1 cell lines. Among the tested compounds, Peniciversiol A showed significant cytotoxic effects against BIO-87 cells (IC50 value 10.21 μM). Penicilactones A-B, Decumbenone A-B, Aspermutarubrol 3-hydroxy-5-(3-hydroxy-5-methylphenoxy)benzoic acid, Cyclopenol, and Violaceol-II showed cytotoxic activity against BEL-7402, BIU-87, and ECA109 cancer cell lines (IC50 values ranging from 7.70 to 20 μM). The anticancer effect of taxol produced by *P. chrysogenum* was tested against several cell lines such as liver cancer cells (HEPG2) and breast adenocarcinoma (MCF7) cells. Results showed that the viability of both cancer cell lines decreased in a dose-dependent manner with an IC50 values of 3.7 and 3.3 μM for HEPG2 and MCF7, respectively [124]. Penichryfuran A exhibited high cytotoxicity against the HepG2 cell line with an IC50 value of 9.0 μM. The ethyl acetate extract of *P. chrysogenum* and Kojic acid showed strong activity against HEP-2 larynx carcinoma cells, with IC50 22.6 ± 0.8 and 23.4 ± 1.4 µg/mL, respectively [125,126]. According to Al-Saleem et al. [126], Kojicacid showed a potent antioxidant activity with IC50m33.7 ± 0.8 µg/mL as revealed by the DPPH free radical-scavenging technique.

### 2.22. Fusarium incarnatum *(Desm.) Sacc.*

*Fusarium incarnatum* is a pathogenic mold belonging to *Nectriaceae*. It is widespread in subtropical and temperate regions as the agent of several crop diseases and mycotoxin producers such as trichothecenes and zearalenone [127]. In freshwater habitats, it was isolated as an endophyte from the roots and leaves of *P. australis*. Very little research has been reported on the medicinal properties of *F. incarnatum*. The ethyl acetate extract of *F. incarnatum* was tested by Das et al. [128] for its antioxidant and antibacterial properties. The fungal extract showed high radical scavenging activity both in ABTS (17.5 ± 0.2 mg TE/g dry extract) and the DPPH assay (IC50 value 379.98 ± 0.8 μg/mL); high reducing power (38.3 ± 0.6 mg AA/g dry extract) and high inhibition capacity of lipid peroxidation (IC50 534.69 ± 2.7 μg/mL). Regarding the antimicrobial effect, it showed inhibition properties against *P. aeruginosa*, *S. aureus*, *E. coli*, *Enterobacter aerogenes*, and *K. pneumoniae* with a diameter of inhibition zone of 15.0 ± 0.9, 12.5 ± 0.5, 16.9 ± 0.2, 15.6 ± 0.7, 10.0 ± 0.1 mm, respectively. Chua et al., 2024 [129] separated bioactive compounds with antimicrobial activity from the crude extract of *F. incarnatum*, showing that Fraction 1 and Fraction 2, rich in alkaloids, terpenoids, flavonoids, and phenolic compounds, had an inhibitory effect against *B. cereus* and *Ganoderma boninense* Pat. with MIC values of 0.156 and 0.3125 mg/mL, respectively.

## 3. Conclusions

**Figure 2:**
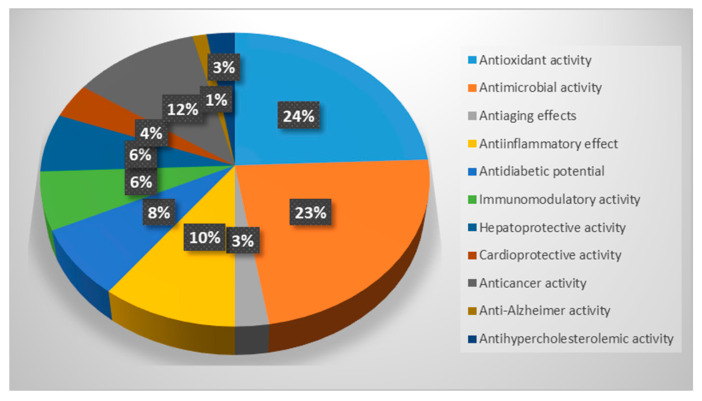
Percentages of freshwater fungi with mentioned medicinal properties.

This review demonstrated that freshwater fungi possess significant medicinal potential. As reported in Figure 2, most of them are known to have antioxidant properties, acting against free radical and cellular oxidation processes, and antimicrobial activity against several pathogenic bacteria and fungi of clinical importance. Among the medicinal properties, antiaging effect, antidiabetic potential, anti-inflammatory, immunomodulatory, hepatoprotective, cardioprotective, anticancer, anti-Alzheimer, and antihypercholesterolemic activities were also reported, confirming that this ecological fungal group represents not only an important part of the wide fungal diversity but also a valid alternative source of bioactive compounds useful for human and animal health.

## Figures and Tables

**Table 1:** Riparian habitats, ecological role, and medicinal properties of freshwater macromycetes observed in previous study by Mirabile et al., 2023 [9].

Taxa	Riparian Habitat	Ecological Categories	Medicinal Properties	References
*Amanita vaginata* (Bull.) Lam.	Mixed forest of *Alnus cordata* (Loisel.) Duby, *Carpinus betulus* L., *Fraxinus ornus* L.	Ectomycorrhizal	Antioxidant activity; ferrous ion chelating ability; antimicrobial activity; esterolytic activity	[11,12,13]
*Armillaria mellea* (Vahl) P. Kumm.	Stumps of *Populus alba* L., *Salix alba* L., *Tamarix africana* Poir., *Alnus cordata*	Necrotroph parasite	Immune regulation; tumor inhibition; antioxidant activity; anti-aging effects; hypoglycemic effect; antimicrobial activity; anti-inflammatory effect; antidiabetic potential; liver protection; reducing DNA damage; anti-Alzheimer activity	[14,15,16,17,18,19,20,21,22,23]
*Armillaria tabescens* (Scop.) Emel.	Stumps of *Populus alba*, *Salix alba*	Necrotroph parasite	Antioxidant activity; metal chelating activity	[24,25,26]
*Astraeus hygrometricus* (Pers.) Morgan	Mixed forest of *Tamarix africana*, *Salix alba*, *Populus alba*	Terricolous saprotroph	Antioxidant, antimicrobial, antiparasitic, anti-diabetic, immunomodulatory, hepatoprotective, anti-inflammatory, cardioprotective, anticancer, chemopreventive activities	[27,28,29,30,31,32,33,34,35,36,37,38,39,40,41,42,43,44,45,46]
*Auricularia auricula-judae* (Bull.) Quél.	Stumps and trunks of *Populus alba*, *Salix alba*	Saprotroph on wood	Skin wound-healing potential; antimicrobial, antioxidant, antidiabetic, anti-inflammatory, anti-obesity, anticancer, anti-radiation, immunomodulatory, hypolipidemic, anticoagulant activities;	[47,48,49,50,51,52,53,54]
*Bjerkandera adusta* (Willd.) P. Karst.	Stumps and trunks of *Populus alba*, *Salix alba*	Necrotroph parasites	Antimicrobial and antioxidant activities	[55,56,57,58,59]
*Bovista nigrescens* Pers.	Mixed forest of *Salix alba*, *Populus alba*, *Eucalyptus camaldulensis Dehnh*.	Saprotroph on litter	Antimicrobial and antioxidant activities	[60,61]
*Calocybe gambosa* (Fr.) Donk	Mixed forest of *Salix alba*, *Populus alba*	Terricolous saprotroph	Antioxidant activity	[62,63,64]
*Candolleomyces candolleanus* (Fr.) D. Wächt. & A. Melzer	Stumps of *Populus alba*, *Salix alba*, *Tamarix africana*, *Alnus cordata*	Saprotroph on wood	Antioxidant, antimicrobial, antiproliferative activities	[65,66,67,68]
*Collybia dryophila* (Bull.) P. Kumm. *Gymnopus dryophylus* (Bull.) Murr	Mixed forest of *Alnus cordata*, *Pinus nigra* J. F. Arnold, *Castanea sativa* Mill., *Salix alba*, *Populus alba*	Saprotroph on litter	Anti-inflammatory activity	[69]
*Coprinus comatus* (O.F. Müll.) Pers.	Mixed forest of *Salix alba*, *Populus alba*, *Alnus cordata*, *Fraxinus ornus*, *Castanea sativa*	Saprotroph on litter	Antioxidant, anticancer, antiandrogenic, hepatoprotective, acetylcholinesterase inhibitory, antiinflammatory, antidiabetic, antiobesity, antibacterial, antifungal, antinematode, anticancer, and antiviral activities	[70,71,72,73,74,75,76]
*Cyclocybe cylindracea* (DC.) Vizzini & Angelini	Stumps of *Populus alba*, *Salix alba*	Saprotroph on wood	Antioxidant activities	[77,78,79]
*Hypsizygus ulmarius* (Bull.) Redhead	Stumps of *Populus alba*, *Salix alba*	Saprotroph on wood	Antioxidant, anti-inflammatory, antibacterial, antitumor activities	[80,81,82,83,84,85,86,87]
*Inonotus hispidus* (Bull.) P. Karst.	Trunks of *Populus alba*, *Salix alba*	Saprotroph on wood	Antioxidant, anticancer, immunomodulatory, antimicrobial activities	[87,88,89,90,91]
*Lactarius controversus* Pers.	Mixed forest of *Salix alba*, *Populus alba*, *Alnus cordata*, *Fraxinus ornus*, *Castanea sativa*	Ectomycorrhizal	Antioxidant and antimicrobial activities	[92,93]
*Lentinus tigrinus* (Bull.) Fr.	Mixed forest of *Salix alba*, *Populus alba*, *Alnus cordata*, *Fraxinus ornus*, *Castanea sativa*	Saprotroph on wood	Antioxidant, antibacterial, hypoglycaemic activities; Anticancer potential;	[94,95,96,97,98]
*Pleurotus cornucopiae* (Paulet) Quél.	Stumps of *Populus alba*	Saprotroph on wood	Antioxidant, anticancer, immunomodulatory activities	[3,5,99,100,101,102]
*P. ostreatus* (*Jacq.*) P. Kumm.	Stumps of *Populus alba*	Saprotroph on wood	Antioxidant, antimicrobial, antidiabetic, anticancer, anti-infiammatory, immunomodulatory, antihypercholesterolemic, antihypertensive, hepatoprotective, antiaging activities	[3,103,104,105,106,107,108,109,110]
*Schizophyllum commune* (Fr.)	Stumps and trunks of *Salix alba*, *Populus alba*	Saprotroph on wood	Antioxidant, anti-inflammatory, hepatoprotective, antimicrobial activities	[111,112,113,114,115,116]

**Table 2:** Medicinal properties and substrate of isolation of freshwater micromycetes.

Taxa	Substrate of Isolation	Medicinal Properties	References
*Penicillium aculeatum*	Leaves of *Phragmites australis*	Antimicrobial activity	[117,118,119]
*P. chrysogenum*	Roots of *P. australis*	Antifungal, anticancer, antimicrobial, antioxidant activities	[120,121,122,123,124,125,126]
*Fusarium incarnatum*	Leaves and roots of *P. australis*	Antimicrobial and antioxidant activity	[127,128,129]

## Data Availability

No new data were created or analyzed in this study.
